# Editorial: Mental-health-related stigma and discrimination: Prevention, role, and management strategies

**DOI:** 10.3389/fpsyt.2023.1136995

**Published:** 2023-01-24

**Authors:** Mohammadreza Shalbafan, Samer El Hayek, Renato de Filippis

**Affiliations:** ^1^Mental Health Research Center, Psychosocial Health Research Institute (PHRI), Department of Psychiatry, School of Medicine, Iran University of Medical Sciences, Tehran, Iran; ^2^Medical Department, Erada Center for Treatment and Rehab in Dubai, Dubai, United Arab Emirates; ^3^Psychiatry Unit, Department of Health Sciences, University Magna Graecia of Catanzaro, Catanzaro, Italy

**Keywords:** community psychiatry, internalized stigma, prejudice, psychiatric disorders, public health, social stigma, stereotyping, stigmatization

Stigmatizing attitudes toward patients suffering from mental illnesses and their caregivers, psychotropic medications, mental health institutions and stakeholders, remain a common public health concern, with major repercussions across countries and cultures worldwide (1). Indeed, a negative attitude toward mental health can lead to avoiding approaching mental health providers, delaying timely diagnosis, poor treatment adherence, and worst disease prognosis and patients' quality of life (2). This phenomenon is particularly evident, but not limited, to patients suffering from mental disorders and has amplified following the COVID-19 pandemic (3–6).

In the Research Topic entitled “*Mental-Health-Related Stigma and Discrimination: Prevention, Role, and Management Strategies*,” we collected 15 articles discussing several aspects of mental-health-related stigma and discrimination from different perspectives and countries, with a particular focus on strategies tackling them. Our editorial aims to summarize their key-points and invite the audience to read this collection.

Several articles highlight the role of psychiatric stigma amongst medical students from various perspectives and how this negative attitude could impact patients with mental illness, particularly those affected by challenging diseases such as schizophrenia spectrum disorders. Movahedi et al. assessed the attitudes of Iranian specialty trainees, who are frontliners dealing with this group of patients, toward providing psychiatric services to patients and their families. They conclude that internal medicine and cardiology residents have more stigmatizing attitudes while psychiatric residents show a more positive behavior toward their patients. In another study conducted in Iran, Zare-Bidaki et al. evaluated the effect of virtual reality on stigma, empathy, and knowledge of medical students toward patients with psychotic disorders. They concluded that this novel tool can be a potential effective instrument in decreasing stigma and increasing empathy and knowledge among medical students (Zare-Bidaki et al.). Moreover, Rezvanifar et al. introduced an educational package for improving the attitude of medical students toward patients with mental disorders based on a scoping review and an expert consensus conducted through a Delphi panel. The developed package contained four interactive interventions: (1) showing a movie and discussing it, (2) implementing psychiatric training including contact with patients living with psychiatric disorders, (3) adopting social communication with patients with psychiatric disorders, and (4) setting up a group discussion on defining stigma and personal experiences (Rezvanifar et al.). Moreover, Mohebbi et al. performed a systematic review to determine Eastern Mediterranean (EMR) medical students' attitudes toward psychiatry, concluding that EMR medical students generally have positive attitudes and predispositions toward the field. On the other hand, Porfyri et al. studied stigma among Greek healthcare professionals and reported that, despite the high level of familiarity, the employees displayed a rather poor willingness to interact with patients with mental illness, and endorsed significant prejudice toward them.

Another group of articles investigated mental-health-related stigma among some influential groups in societies, and which interventions may improve their attitudes toward patients. Taghva et al. performed a two-day training workshop to improve the attitude of clergymen toward patients with mental disorders. Findings showed that the awareness and attitude of ecclesiastics toward mental health and its consequential stigma were relatively good, and significantly improved upon holding the workshop (Taghva et al.). In addition, Eissazade et al. investigated the attitude of a group of Iranian theater artists toward patients with mental disorders, as well as their own mental health. Participants' strongest fears were to allow an individual with a severe mental disorder to take care of their children and the possibility of patients in this group to obtain a hunting license. Twenty-five percent of participants were at risk of moderate to severe anxiety, and 17.3% participants were at risk of moderate to severe depression (Eissazade et al.).

Several other articles investigated stigma among general populations, as well as some strategies to fight this issue. Ruiz et al. reported findings of a survey on stigma among university students in Valencia, Spain. They found that women show fewer stigmatizing attitudes than men but similar stereotypes and prejudice toward people with mental disorders. The survey as also found students of medicine, psychology, and teaching to have fewer stigmatizing attitudes than students of economics and data science, but differences between degrees were more subtle in terms of stereotypes and prejudice toward people with mental disorders (Ruiz et al.). In an opinion piece, Saboury Yazdy et al. shared their experience using a smartphone application called “Be my Voice” to break social stigma against domestic violence in Iran. Sawaguchi et al. reported their findings on COVID-19-related stigma and its relationship with mental wellbeing using a cross-sectional analysis of a cohort study in Japan. They concluded that people aged ≥ 70 years are more likely to exhibit COVID-19-related stigma. Additionally, the results indicate that COVID-19-related stigma negatively impacts quality of life secondary to the underlying psychological distress (Sawaguchi et al.).

Two other articles focused on the relationship between stigma and suicide as a major public mental health problem. In a perspective piece, Shoib et al. raised concerns on the relationship between suicide, stigma, and COVID-19 in low- and middle-income countries. The article particularly addresses the potential link between social stigma and suicide in the wake of the current coronavirus pandemic and proposes some practical ideas for reducing mental-health-related stigma (Shoib et al.). Masoomi et al. in another perspective piece, also raised the issue of stigma as a barrier to suicide prevention strategies in Iran.

Two other publications evaluated psychometric properties of stigma-related questionnaires. Dinmohammadi et al. evaluated psychometric properties of the Self-Stigma Inventory for Iranian families of persons who use drugs, and concluded that it is a valid and reliable scale with three factors and 14 items. Additionally, Burzee et al. re-evaluated Stuart's Police Officer Stigma Scale and their findings imply that this scale is reliable but needs to include two components rather than one.

Last but not the least, de Filippis et al. performed a clinical study on internalized-stigma and dissociative experiences in a sample of patients affected by bipolar disorder in a clinical outpatient setting in Catanzaro, Italy. Their findings suggest that self-stigma is associated with dissociative symptoms, reducing overall quality of life. Thus, authors recommended the early identification of at-risk patients with previous lifetime abuse and high perceived stigma, which could lead the way for an ever more precise tailoring of treatment management in bipolar disorder (de Filippis et al.).

All in all, the articles collected in this Research Topic reemphasize the importance of mental-health-related stigma as a major public health issue. Due to ongoing substantial research in this field, “*Community Series in Mental-Health-Related Stigma and Discrimination: Prevention, Role, and Management Strategies – Volume II*” has been launched for further submissions and we are looking forward to continue exploring this topic.

## Author contributions

All authors listed have made a substantial, direct, and intellectual contribution to the work and approved it for publication.
